# Selective CB_2_ Receptor Agonist, HU-308, Reduces Systemic Inflammation in Endotoxin Model of Pneumonia-Induced Acute Lung Injury

**DOI:** 10.3390/ijms232415857

**Published:** 2022-12-13

**Authors:** Stefan Hall, Sufyan Faridi, Purvi Trivedi, Saki Sultana, Bithika Ray, Tanya Myers, Irene Euodia, David Vlatten, Mathieu Castonguay, Juan Zhou, Melanie Kelly, Christian Lehmann

**Affiliations:** 1 Department of Physiology and Biophysics, Dalhousie University, Halifax, NS B3H 1X5, Canada; 2 Department of Anesthesia, Pain Management and Perioperative Medicine, Dalhousie University, Halifax, NS B3H 4R2, Canada; 3 Department of Pharmacology, Dalhousie University, Halifax, NS B3H 4R2, Canada; 4 Department of Ophthalmology & Visual Sciences, Dalhousie University, Halifax, NS B3H 4R2, Canada; 5 Department of Pathology, Dalhousie University, Halifax, NS B3H 4R2, Canada; 6 Department of Microbiology and Immunology, Dalhousie University, Halifax, NS B3H 4R2, Canada

**Keywords:** acute lung injury, ARDS, inflammation, cytokines, cannabinoid type II receptor (CB_2_), intravital microscopy, microcirculation

## Abstract

Acute respiratory distress syndrome (ARDS) and sepsis are risk factors contributing to mortality in patients with pneumonia. In ARDS, also termed acute lung injury (ALI), pulmonary immune responses lead to excessive pro-inflammatory cytokine release and aberrant alveolar neutrophil infiltration. Systemic spread of cytokines is associated with systemic complications including sepsis, multi-organ failure, and death. Thus, dampening pro-inflammatory cytokine release is a viable strategy to improve outcome. Activation of cannabinoid type II receptor (CB_2_) has been shown to reduce cytokine release in various in vivo and in vitro studies. Herein, we investigated the effect of HU-308, a specific CB_2_ agonist, on systemic and pulmonary inflammation in a model of pneumonia-induced ALI. C57Bl/6 mice received intranasal endotoxin or saline, followed by intravenous HU-308, dexamethasone, or vehicle. ALI was scored by histology and plasma levels of select inflammatory mediators were assessed by Luminex assay. Intravital microscopy (IVM) was performed to assess leukocyte adhesion and capillary perfusion in intestinal and pulmonary microcirculation. HU-308 and dexamethasone attenuated LPS-induced cytokine release and intestinal microcirculatory impairment. HU-308 modestly reduced ALI score, while dexamethasone abolished it. These results suggest administration of HU-308 can reduce systemic inflammation without suppressing pulmonary immune response in pneumonia-induced ALI and systemic inflammation.

## 1. Introduction

Bacterial and viral pneumonias represent a major cause of critical illness. Among other complications, patients hospitalized with pneumonia are at high risk for inflammatory lung injury termed acute respiratory distress syndrome (ARDS), which carries a mortality rate of approximately 40% [1,2,3]. Additionally, over one-third of these patients may develop sepsis [4,5], a severe systemic inflammatory response associated with multi-organ failure and high mortality [6]. Patients who survive the acute phase remain at risk for long-term sequelae and long-term mortality, thus compounding the disease burden [7]. Furthermore, this burden has been exacerbated in recent times by influx of COVID-19 patients, approximately 5% of whom have been reported to develop critical disease characterized by ARDS, septic shock, or multi-organ failure [8]. To date, there remains no specific highly effective therapy for pneumonia-induced ARDS or sepsis [4,5,9].

The pathophysiology underlying ARDS is generally termed acute lung injury (ALI) [9] and is heavily derived from pulmonary innate immune responses [10]. During pulmonary infection, pattern recognition receptors (PRRs) in alveolar epithelial cells and resident macrophages are stimulated by pathogen-associated molecular patterns (PAMPs). Subsequent signaling cascades lead to local release of pro-inflammatory cytokines [11] which promote further recruitment of circulating immune cells, predominantly neutrophils, to assist in anti-microbial defense [1]. While these processes are essential for local pathogen clearance, excessive and unresolved inflammatory signaling contributes to a breakdown of pulmonary epithelial and endothelial barrier integrity, alveolar edema, and impaired gas exchange [11]. Indeed, numerous studies report elevated pro-inflammatory cytokine levels as a consistent feature of ARDS [12,13]. Higher levels of these cytokines in the systemic circulation have been associated with mortality [14,15,16]. Therapies aimed at dampening pro-inflammatory cytokine release may thus be a viable strategy to improve outcome.

Numerous studies have demonstrated the immunomodulatory capacity of the endocannabinoid system. Pharmacological activation of the cannabinoid type II receptor (CB_2_) has been shown to reduce pro-inflammatory cytokine release in various in vivo and in vitro studies, as reviewed by Turcotte and colleagues [17]. CB_2_ is family A G protein-coupled receptor which is highly expressed in peripheral immune tissues and leukocyte populations [18]. Agonist activation of CB_2_ causes dissociation of Gαi/o and Gβγ subunits from the receptor [19] and leads to various downstream outcomes which exert effects on cytokine transcription, such as decreased DNA-binding of cAMP response element binding (CREB) transcription factors [20] and increased p38 mitogen-activated protein kinase (MAPK) signaling [21]. Moreover, numerous studies have demonstrated a beneficial role for CB_2_ in experimental animal models of disease. Synthetic agonists of CB_2_ have been shown to ameliorate lung injury and improve survival in experimental sepsis [22,23,24,25]. Furthermore, pharmacological modulation of other endocannabinoid mediators in LPS-induced ALI has supported a lung-protective role for CB_2_ [26,27].

In this study, we explored therapeutic effects of CB_2_ activation in a murine model of ALI-induced systemic inflammation. We hypothesized that treatment with HU-308, a highly specific agonist of CB_2_ [28], reduces systemic inflammation by modulation of pro-inflammatory cytokine release. To compare efficacy with a known positive control, we employed the synthetic glucocorticoid dexamethasone (DEX), an immunosuppressant which exerts potent anti-inflammatory effects via the glucocorticoid receptor and which has been used in the treatment of COVID-19-related ARDS [29].

## 2. Results

### 2.1. Intranasal Endotoxin Induces Acute Lung Injury and Systemic Inflammation

To recapitulate key aspects of ALI, we employed a murine model of intranasal endotoxin (lipopolysaccharide—LPS) administration and assessed pulmonary and systemic inflammatory responses. Tissues were collected at 2, 4, and 6 h post-administration to identify an optimal timepoint of elevated systemic cytokine release.

#### 2.1.1. Intranasal Endotoxin Induces Acute Lung Injury

**Figure 1:**
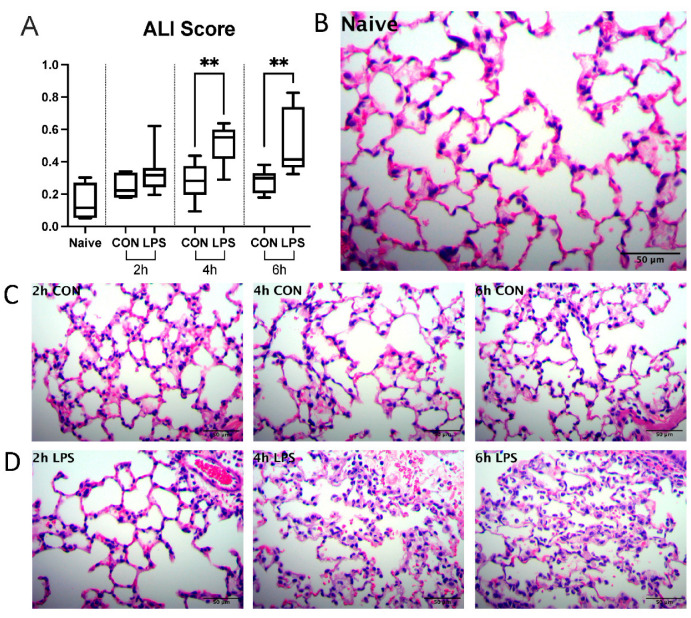
Intranasal administration of LPS from P. aeruginosa induces ALI at 4, 6 h post-induction. (**A**) Histopathological lung scores. (**B**) Representative fields of view (FOV) from naïve group. (**C**) Representative FOV from control (CON) group at 2, 4, and 6 h timepoints. (**D**) Representative FOV from LPS group at 2, 4, and 6 h timepoints. Whiskers show minimum and maximum values, boxes represent 25–75% data ranges, and horizontal lines within boxes represent medians. Statistically significant differences were determined via Mann–Whitney tests between LPS and control groups at each timepoint and are depicted as ** *p* ≤ 0.01.

Hematoxylin and eosin (H&E)-stained right lung sections from LPS-challenged mice (Figure 1A) exhibited greater evidence of neutrophil infiltration, alveolar edema, and interstitial thickening versus naïve (Figure 1B) or control (CON) mice (Figure 1C). LPS-challenged mice had significantly higher lung injury scores at 4, and 6 h timepoints versus respective controls (Figure 1D). Notably, hyaline membranes, a pathological component of human ALI rarely observed in murine models, were not observed in our model.

#### 2.1.2. Intranasal Endotoxin Administration Upregulates Plasma Cytokines

**Figure 2:**
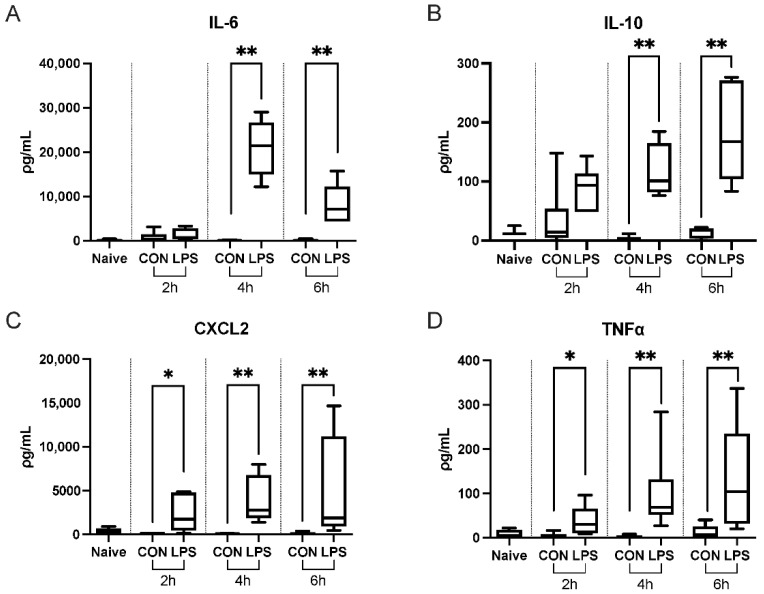
Systemic upregulation of cytokines following ALI. Plasma levels of (**A**) IL-6, (**B**) IL-10, (**C**) CXCL2, and (**D**) TNFα were elevated at 4 and 6 h post-induction. Whiskers show minimum and maximum values, boxes represent 25–75% data ranges, and horizontal lines within boxes represent medians. Statistically significant differences were determined via Mann–Whitney tests between LPS and control groups at each timepoint and are depicted as * *p* ≤ 0.05, ** *p* ≤ 0.01.

Using a multiplex bead-based assay, we assessed cytokine levels in mouse plasma samples from all groups. Interleukin-6 (IL-6) was significantly elevated at 4 and 6 h post induction in LPS-challenged mice versus respective controls (Figure 2A). Interleukin-10 (IL-10) demonstrated significant elevations in the same manner (Figure 2B). Chemokine (C-X-C motif) ligand 2 (CXCL2), was significantly elevated in all LPS-challenged groups versus respective controls, however this elevation was higher in 4 and 6 h groups versus 2 h (Figure 2C). Tumor necrosis factor α (TNFα) was significantly elevated in LPS-challenged groups in a similar manner. These data indicate systemic inflammatory response to intranasal LPS administration at 4 and 6 h post-induction. Based on overall trends, 6 h was selected as the timepoint for future experimentation.

### 2.2. CB_2_ Agonist Reduces Systemic Inflammation

Using the selected 6 h timepoint, we examined the effect of HU-308, a CB_2_-specific cannabinoid receptor agonist, on plasma cytokine levels and peripheral (intestinal) microcirculatory parameters. A 3 mg/kg dose (i.v.) was selected based on earlier studies in the Lehmann laboratory [24]. The efficacy of HU-308 was compared to DEX (0.1 mg/kg, i.v.).

#### 2.2.1. CB_2_ Agonist Reduces Systemic Cytokine Release

**Figure 3:**
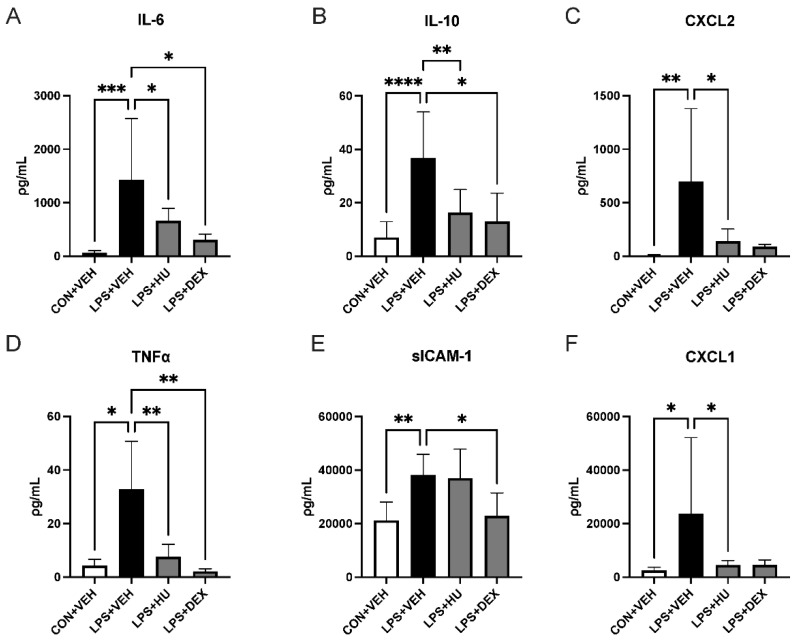
Impact of treatment on ALI-induced systemic cytokine release. Plasma levels of (**A**) IL-6, (**B**) IL-10, (**C**) CXCL2, (**D**) TNFα, (**E**) sICAM-1, and (**F**) CXCL1 were reduced following administration of HU-308 or DEX. Data are expressed as mean ± SD. Statistically significant differences were determined via one-way ANOVA with Dunnett’s multiple comparisons test and are depicted as * *p* ≤ 0.05, ** *p* ≤ 0.01, *** *p* ≤ 0.001, **** *p* ≤ 0.0001.

Consistent with initial data, LPS-challenged mice with vehicle treatment (LPS + VEH) exhibited marked elevations in plasma cytokines including IL-6, IL-10, CXCL2, TNFα, soluble intercellular adhesion molecule-1 (sICAM-1), and chemokine (C-X-C motif) ligand 1 (CXCL1) versus control animals (CON + VEH; Figure 3A–D). HU-308 significantly reduced plasma levels of all mediators except sICAM-1. DEX significantly reduced sICAM-1 and appeared to reduce levels of all other pro-inflammatory mediators.

#### 2.2.2. CB_2_ Agonist Improves Intestinal Microcirculation Impaired by ALI

**Figure 4:**
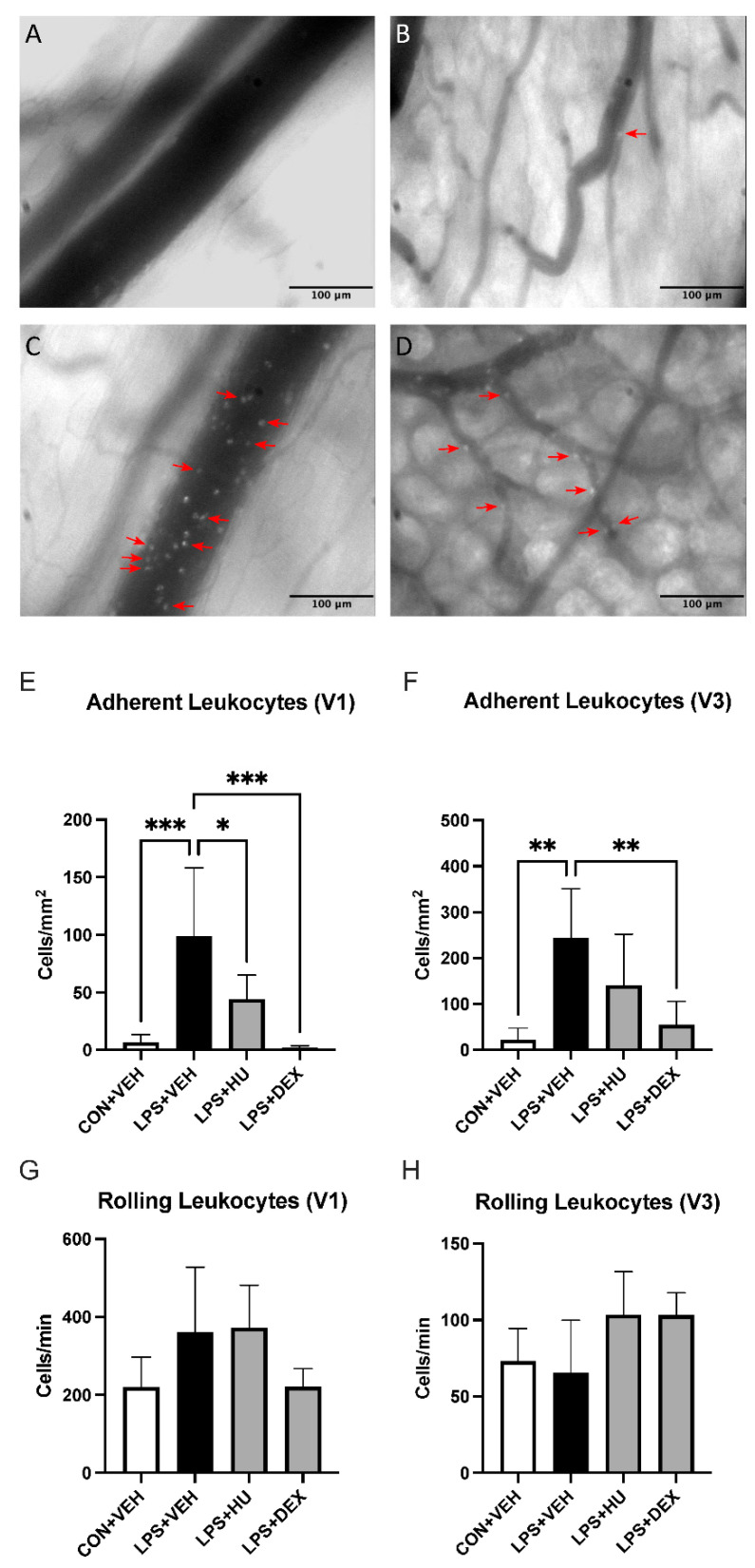
Impact of LPS and treatment on intestinal leukocyte activation. Representative images of ileal submucosal (**A**) V1 and (**B**) V3 venules in con + veh mice, and (**C**,**D**) lps + veh mice. Red arrows denote adherent leukocytes. Analysis of leukocyte adhesion in (**E**) V1 and (**F**) V3 venules. Analysis of leukocyte rolling in (**G**) V1 and (**H**) V3 venules. Data are expressed as mean ± SD. Statistically significant differences were determined via one-way ANOVA with Dunnett’s multiple comparisons test and are depicted as * *p* ≤ 0.05, ** *p* ≤ 0.01, *** *p* ≤ 0.001.

LPS-challenged mice exhibited a marked increase in number of adherent leukocytes per endothelial area in collecting (V1) and post-capillary (V3) venules of the ileal microvasculature. HU-308 attenuated this increase in V1 venules and appeared to exert a similar effect in V3 venules. DEX abolished leukocyte adhesion in V1 venules and markedly reduced it in V3 venules, while combination treatment exhibited a slightly less potent effect. Inter-group differences were also observed in leukocyte rolling; however, these differences were not statistically significant (Figure 4, Videos S1–S4).

**Figure 5:**
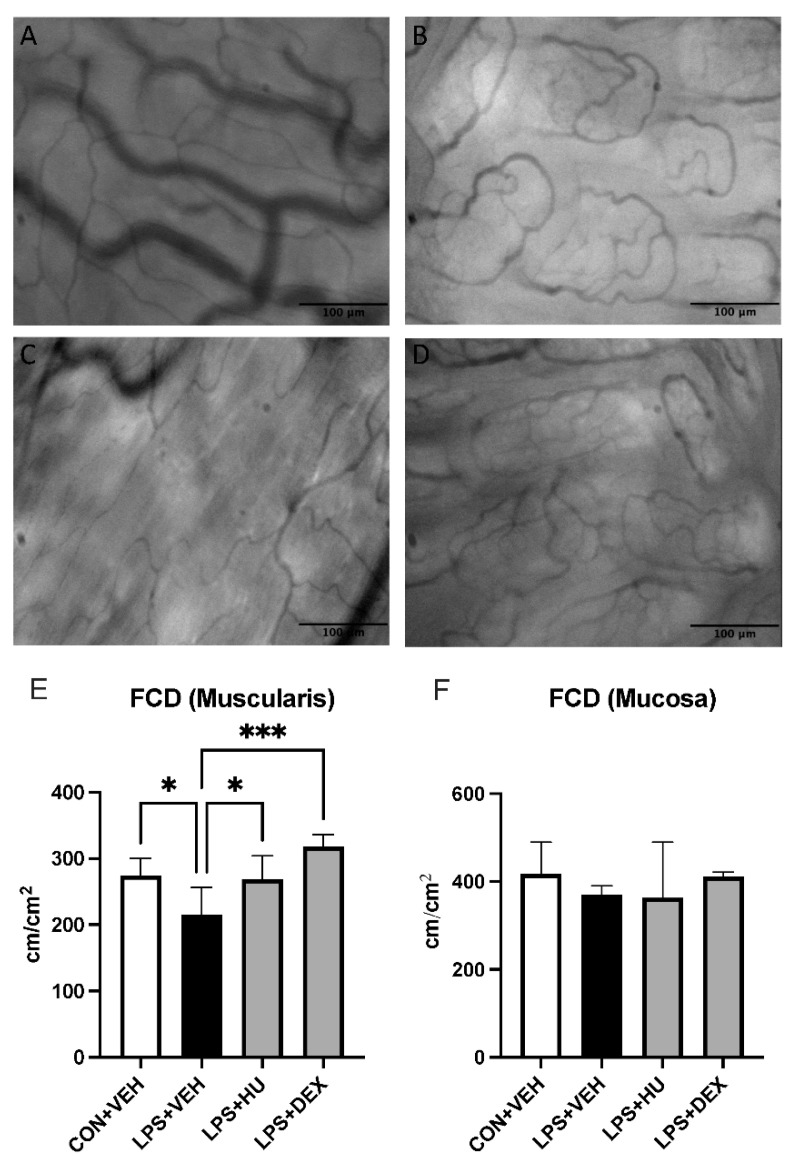
Impact of LPS and treatment on intestinal capillary perfusion. Representative images of ileal (**A**) muscularis and (**B**) mucosal capillaries in con + veh mice, and (**C**,**D**) LPS + veh mice. Analysis of FCD in (**E**) muscularis and (**F**) mucosal layers. Data are expressed as mean ± SD. Statistically significant differences were determined via one-way ANOVA with Dunnett’s multiple comparisons test and are depicted as * *p* ≤ 0.05, *** *p* ≤ 0.001.

We assessed inter-group differences in capillary perfusion by calculating functional capillary density (FCD) in the intestinal mucosa and muscularis. Muscularis FCD was significantly impaired in LPS-challenged mice. HU-308 rescued FCD and DEX improved FCD above baseline. Analysis of mucosal FCD yielded no statistically significant differences (Figure 5, Videos S5–S8).

### 2.3. CB_2_ Agonist Reduces Pulmonary Inflammation

Infiltration of pulmonary tissues by inflammatory immune cells, especially neutrophils, is a crucial component of host defense but also contributes to ALI/ARDS. Here, we studied the effect of HU-308 on LPS-induced pulmonary inflammation via histological and IVM approaches.

#### 2.3.1. CB_2_ Agonist Modestly Reduces ALI

**Figure 6:**
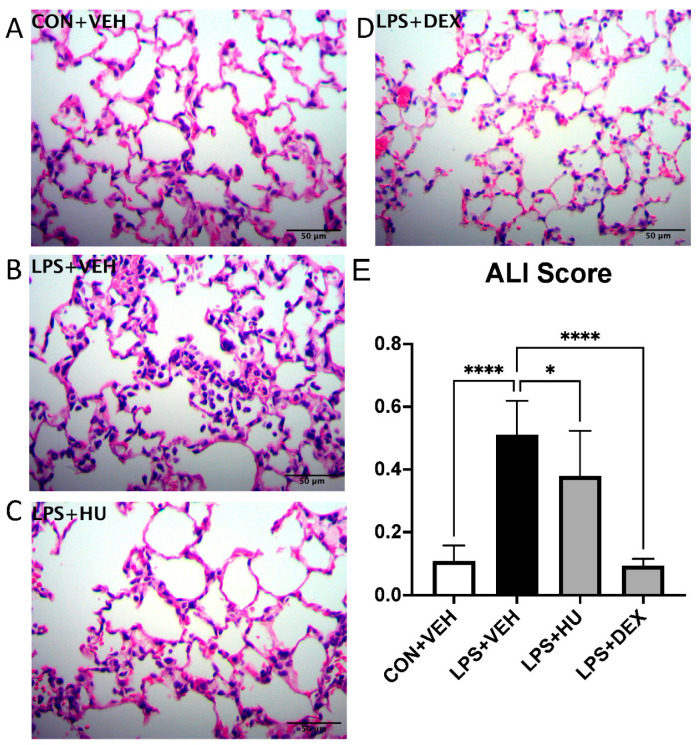
Impact of treatment on LPS-induced lung histopathology. Representative FOV of (**A**) con + veh and LPS + (**B**) veh, (**C**) HU-308, and (**D**) DEX mice. (**E**) Histopathological lung scores. Data are expressed as mean ± SD. Statistically significant differences were determined via one-way ANOVA with Dunn’s multiple comparisons test and are depicted as * *p* ≤ 0.05, **** *p* ≤ 0.0001.

Using the ALI score algorithm, we compared inter-group differences in lung histopathology at 6 h post-induction. As expected, LPS-challenged mice demonstrated a robust increase in ALI score vs. control (Figure 6). HU-308 modestly but significantly reduced ALI score, suggesting retention of immune activation. ALI score was nearly abolished by DEX, suggesting a strong immunosuppressive effect.

#### 2.3.2. Intranasal Endotoxin and CB_2_ Agonist Impact Pulmonary Microcirculation

**Figure 7:**
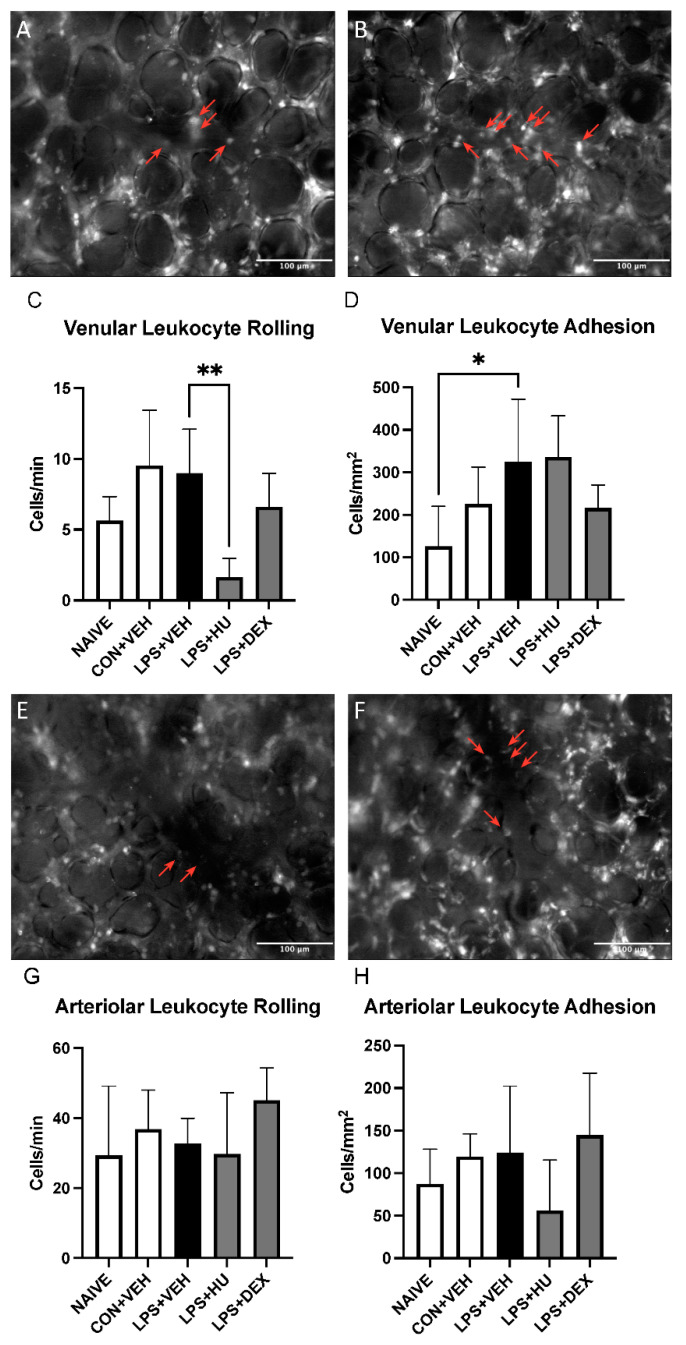
Impact of LPS and treatment on leukocyte activation in pulmonary venules and arterioles. Representative images of pulmonary venules in (**A**) con + veh mice and (**B**) LPS + veh mice. Analysis of leukocyte (**C**) rolling and (**D**) adhesion in pulmonary venules. Representative images of pulmonary arterioles in (**E**) con + veh mice and (**F**) LPS + veh mice. Analysis of leukocyte (**G**) rolling and (**H**) adhesion in pulmonary arterioles. Red arrows denote adherent leukocytes. Data are expressed as mean ± SD. Statistically significant differences were determined via one-way ANOVA with Dunnett’s multiple comparisons test and are depicted as * *p* ≤ 0.05, ** *p* ≤ 0.01.

Venular leukocyte adhesion was increased in LPS-challenged mice versus naïve animals, but not versus other groups. Venular leukocyte rolling, arteriolar leukocyte adhesion, and arteriolar leukocyte rolling were not significantly increased in LPS-challenged versus naïve or control mice. HU-308 significantly reduced venular leukocyte rolling vs. LPS-challenged mice, although this did not represent restoration of control values (Figure 7, Videos S9–S12).

**Figure 8:**
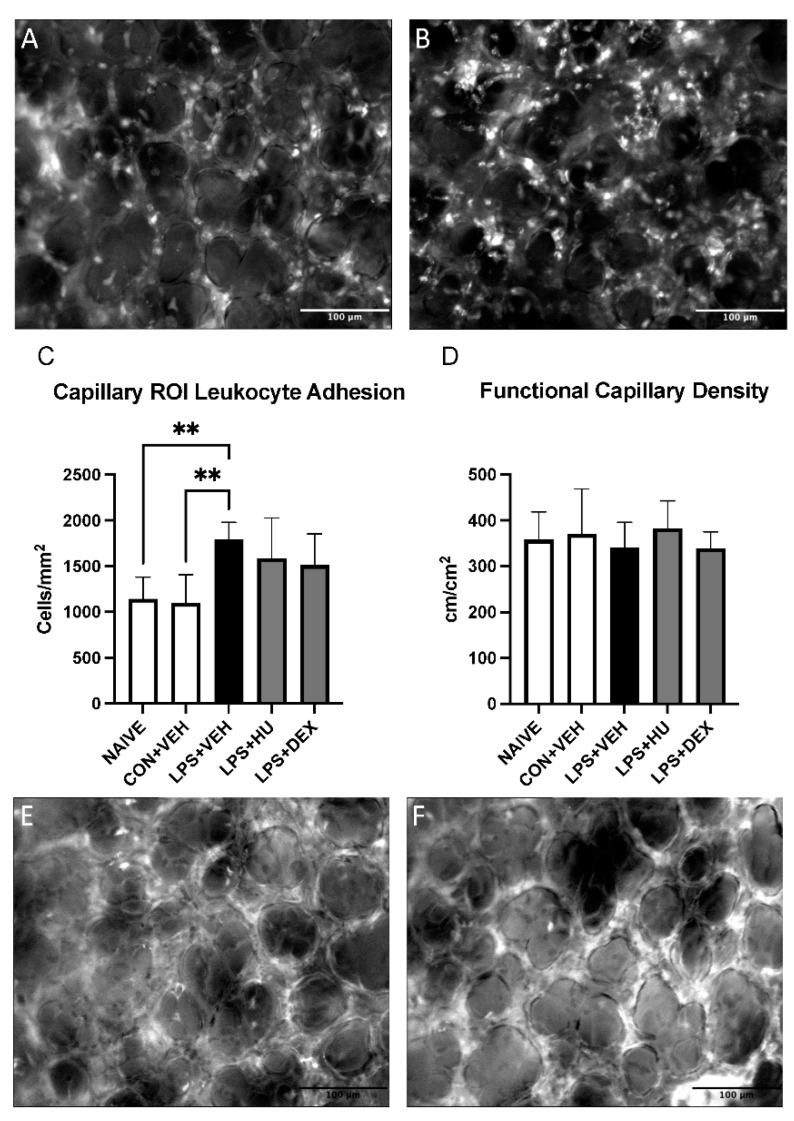
Impact of LPS-induced ALI and treatment on pulmonary capillaries. Representative images of leukocyte adhesion in capillary ROI in (**A**) con + veh mice and (**B**) LPS + veh mice. (**C**) Analysis of leukocyte adhesion in capillary ROI. (**D**) Analysis of FCD in capillary ROI. Representative images of FCD in capillary ROI in (**E**) con + veh mice and (**F**) LPS + veh mice. Data are expressed as mean ± SD. Statistically significant differences were determined via one-way ANOVA with Dunnett’s multiple comparisons test and are depicted as ** *p* ≤ 0.01.

Capillary region-of-interest (ROI) leukocyte adhesion was significantly elevated in LPS mice versus both naïve and control mice. Treatment with HU-308 or DEX did not significantly reduce leukocyte adhesion in LPS-challenged mice. Despite alterations in leukocyte adhesion, no statistically significant differences in FCD were observed (Figure 8, Videos S13–S16).

## 3. Discussion

While CB_2_ activation has been previously shown to exert anti-inflammatory effects in numerous models of disease, fewer studies have explored its therapeutic potential in ALI [22,23]. Herein, we present the first study employing a specific CB_2_ agonist in a model of ALI-induced systemic inflammation. Through molecular, histological, and intravital methods, we demonstrate pulmonary and systemic anti-inflammatory effects of the synthetic CB_2_ agonist, HU-308.

We observed a significant increase in lung histopathology injury scores following intranasal administration of LPS from P. aeruginosa at 4 and 6 h post-induction. Similar timepoints have been demonstrated in previous studies employing LPS from common respiratory pathogens, *P. aeruginosa* and *E. coli* [30,31]. Notably, intranasal saline administration was associated with a minor increase in lung histopathology vs. naïve (Figure S1). We attribute this to wash-down of contaminants from the upper respiratory tract into the lower respiratory tract leading to stimulation of immune response. Importantly, however, intranasal saline administration was only associated with minor systemic release of two of six reported inflammatory mediators (Figure S2).

Intranasal LPS administration also induced significant increases in plasma IL-6, IL-10, CXCL2, and TNFα coincident with onset of ALI. Systemic pro-inflammatory cytokine release is not often studied in endotoxin models of ALI but has been reported in a Poly(I:C) model of COVID-19-induced ALI [32]. Nonetheless, numerous observational clinical studies report this phenomenon in pneumonia and pneumonia-induced ALI. In one such example, a prospective study by Fernandez-Botran et al., reported elevated IL-6, IL-10, and TNFα in severe community-acquired pneumonia (CAP) vs. non-severe CAP [15]. More recent reports have associated the same pro-inflammatory mediators with mortality in severe COVID-19 [33]. Therefore, our selected model bears important similarity to the clinical picture of pneumonia-induced ALI.

HU-308 significantly reduced systemic levels of IL-6, IL-10, CXCL2, TNFα, and CXCL1 in LPS-challenged mice. HU-308 has been reported to reduce plasma CXCL1, CXCL2, and TNFα in rodent models of sepsis [24], hepatic injury [34], and nephropathy [35]. JWH-133, which also selectively activates CB_2_, was shown to reduce serum IL-6 and TNFα at 24 h post-induction in cecal ligation and puncture (CLP)-induced sepsis [25]. The same study also reported that intraperitoneal administration of JWH-133 increased serum levels of IL-10. JWH-133 has been demonstrated to increase IL-10 production in vitro in murine peritoneal macrophages [36], a cell population which would be susceptible to intraperitoneal administration. Intravenous administration of CB_2_ agonist, as in our model, could theoretically impact other innate immune cell populations such as circulating neutrophils, particularly those marginated within the lung microvasculature [37]. By binding with CB_2_ expressed on these neutrophils, HU-308 could reduce DNA-binding of CREB and subsequently reduce IL-10 production [38], thus leading to a decrease in plasma IL-10.

Using IVM, we observed a robust increase in leukocyte adhesion in V1 and V3 venules of LPS-challenged mice. HU-308 significantly reduced leukocyte adhesion in V1 and appeared to exert a similar effect in V3 venules. This result is in agreement with a previous study by our group employing 2.5 mg/kg HU-308 and 10 mg/kg HU-308 in models of endotoxemia and sepsis, respectively, and may be suggestive of moderate immunomodulation as opposed to strong immunosuppression [24]. By comparison, DEX abolished and significantly reduced leukocyte adhesion in V1 and V3 venules, respectively. This may be related to sICAM-1, which has been proposed to increase endothelial cell activation [39]. In agreement with this, sICAM-1 was reduced in DEX- but not HU-308-treated mice. Additionally, reductions in leukocyte adhesion by both HU-308 and DEX may also be related to observed reductions in plasma chemokines CXCL1 and CXCL2, which are key players in neutrophil recruitment to inflamed tissues [40].

In addition to increased leukocyte adhesion, our experimental model also exhibited slight impairment of intestinal capillary perfusion. Impaired capillary perfusion contributes to tissue hypoxemia and organ failure in sepsis and has been well described in pre-clinical models [41]. In our experiments, LPS administration significantly reduced FCD in muscularis but not mucosal layers. This suggests relatively minor impairment compared to previously reported models of endotoxemia which report up to 50% or greater reduction in FCD in both muscularis and mucosal layers [42]. Nonetheless, slight reduction in muscularis FCD in our experiments may still be indicative of microcirculatory impairment, and rescue of FCD in treatment groups supports this. In this regard, FCD was rescued by combination treatment and, interestingly, improved above baseline by DEX. Importantly, HU-308 also appeared to rescue muscularis FCD, an outcome which would contrast with previously reported findings in an endotoxemia model [43]. However, this effect did not reach statistical significance, and further studies are required to clarify the role of HU-308 in improving intestinal capillary perfusion.

To study the impact of CB_2_ activation on pulmonary inflammation in our model, we performed a separate histopathological analysis to compare control, LPS, and treatment groups. As in the first iteration, we observed a robust increase in ALI score in LPS-challenged mice. In treatment groups, HU-308 modestly but significantly diminished ALI score, whereas DEX and combination treatment fully suppressed it. This is somewhat distinct from studies on septic lung injury which report stronger reductions in lung histopathology in mice administered with selective CB_2_ agonists. For example, Liu and colleagues reported a near-baseline reduction in lung injury score following treatment with 2.5 mg/kg HU-308, and Çakır and colleagues reported a similar effect following treatment with 5 mg/kg JWH-133 [22,25]. However, sepsis- and pneumonia-induced ALI differ in pathophysiology and thus therapeutic efficacy should be expected to differ. Systemic administration might be most efficacious in a sepsis model, while direct administration to the respiratory tract could theoretically be more efficacious in a pneumonia-induced ALI model. Further research into such questions will depend on development of novel drug formulations to access alternative routes of administration.

Leukocyte trafficking to the lungs is a key component of ALI, so we employed a previously described lung IVM protocol [44] to study leukocyte rolling and adhesion in venules and arterioles. Although two statistically significant differences were identified, a high degree of variability limited interpretation of overall trends in the data. Previous studies have reported elevated leukocyte rolling and adhesion in both pulmonary arterioles and venules following CLP-induced sepsis [45,46]. Relative to these findings, we observed far lower levels of leukocyte adhesion in both vessel types. This may be attributable to the experimental model resulting in a potential mismatch between pulmonary LPS localization following inhalational administration and peripheral lung imaging. In an intranasal model, LPS solution enters the lungs via proximal airways. Conversely, the low penetration depth of conventional fluorescence imaging limits lung IVM to distal airway regions, i.e., the subpleural microvasculature [47]. Thus, it is quite possible that inflammation does not spread to the imaged region, which is therefore not representative of the sought-after immune response. The same logic, however, does not apply to the CLP-induced sepsis model. Rather, inflammation would be expected to spread through the lung as determined by perfusion, thus leading to observable effects in the subpleural microvasculature.

The pulmonary capillaries play crucial roles in gas exchange and immune surveillance, so we used lung IVM to examine leukocyte adhesion and FCD within capillary ROI. We observed overall high levels of capillary leukocyte adhesion as well as marked increases in LPS-challenged mice vs. both naïve and control mice. This finding may speak to the vast marginated pool of neutrophils which reside in the pulmonary capillaries under normal conditions, as described by Yipp and colleagues [37]. It is also consistent with the works of Roller, Wang, and colleagues, which demonstrate a similar trend in a CLP-induced sepsis model [45,46]. HU-308 and DEX appeared to reduce capillary leukocyte adhesion, although further experimentation is required to confirm this trend. A study by Park and colleagues reported that entrapped neutrophils in pulmonary capillaries impair gas exchange through physical blockade of erythrocyte flow [48]. Therefore, we analyzed FCD in capillary ROI, but surprisingly found no impairment in LPS-challenged mice. This may be explicable by lack of inflammation in imaged regions and conflicting findings which report that neutrophils residing in the pulmonary capillaries do not impair perfusion, as reviewed by Neupane and Kubes [49].

There are some limitations in the present study. First, while endotoxin is a powerful stimulator of TLR2- and TLR4-mediated immune responses which play key roles in bacterial pneumonia-induced ALI, it does not fully recapitulate pneumonia caused by an infectious and proliferating pathogen. Further studies should explore the impact of exogenous CB_2_ activation in the context of viable respiratory pathogens to better understand its impact on immunity and inflammation. Second, HU-308 was administered immediately following ALI induction. Treatment at later timepoints, with different doses, and via different routes of administration may result in important differences in outcome, and thus ought to be considered in future experiments. Third, our methods are primarily observational in nature. While the CB_2_-selectivity and downstream effects of HU-308 have been extensively reported [17,28], possibility of off-target effects cannot yet be entirely excluded. Future use of CB_2_ knockout backgrounds and pharmacological antagonists will be important in this regard. Finally, our study employed only 12-week-old male mice. There exist sex and age differences in susceptibility to pneumonia in both humans and mice which should be regarded in interpretation of results [50].

The therapeutic potential of the CB_2_ receptor in pneumonia-induced ALI and systemic inflammation is still understudied. In this study, we discovered that the selective CB_2_ agonist HU-308 significantly reduced LPS-induced systemic inflammation in ALI while modestly reducing pulmonary immune activation. This contrasted with DEX, which suppressed immune responses both at the systemic and pulmonary levels. Mechanisms of this differential response may be related to high localization of CB_2_ on circulating immune cells [17] and remains to be explored in future studies. Importantly, this may be beneficial in the context of pneumonia-induced ALI by preserving host defense in the lungs while limiting peripheral organ damage. This is also supported by clinical data. A prospective observational cohort study indicated that patients with severe CAP fail to mount a robust local inflammatory response, but instead demonstrate a greater systemic inflammatory response compared to non-severe patients [15]. Thus, the results herein suggest potential therapeutic efficacy of CB_2_ activation in improving outcome in pneumonia-induced ALI.

## 4. Materials and Methods

### 4.1. Animals

All experiments were approved by the Dalhousie University Committee on Laboratory Animals (Protocol #20-075). Male C57Bl/6 mice (12 weeks old, 20–30 g) were purchased from Charles River Laboratories (Senneville, QC, Canada). Mice were housed in ventilated, climate-controlled cages at 22 °C on a 12 h light/dark cycle in the Carleton Animal Care Facility (CACF) of the Sir Charles Tupper Medical Building (Halifax, NS, Canada) with ad libitum access to standard rodent chow and water. Mice were acclimatized for a minimum of one week prior to experimentation. All experimental procedures were approved by the University Committee on Laboratory Animals at Dalhousie University under protocol number #20-075 and were performed following the guidelines and standards of the Canadian Council on Animal Care.

### 4.2. Reagents

Lipopolysaccharide (LPS) from Pseudomonas aeruginosa was purchased from Sigma-Aldrich (Oakville, ON, Canada) and dissolved in sterile saline (0.9% NaCl) to a concentration of 10 mg/mL prior to storage at −20 °C. HU-308 was purchased from Tocris Bioscience (Toronto, ON, Canada) and dissolved in anhydrous ethanol at 20 mg/mL prior to preparation in 1:1:18 solution of HU-308: Kolliphor EL: sterile saline and stored at 4 °C. Dexamethasone (Sandoz; Basel, Switzerland) was dissolved in sterile saline to a concentration of 33.3 µg/mL and stored at room temperature. Albumin-fluorescein isothiocyanate (FITC-albumin; λEX = 450–490 nm; Sigma-Aldrich) was dissolved in sterile saline to 50 mg/mL. Rhodamine-6G (λEX = 515–560 nm; Sigma-Aldrich) was dissolved in sterile saline to 0.5 mg/mL. Sodium pentobarbital (240 mg/mL; BimedaMTC Animal Health Inc.; Cambridge, ON, Canada) was diluted with sterile saline and used to provide anesthesia or euthanasia, where applicable. Isoflurane USP (99.9%; Fresenius Kabi; Bad Homburg, Germany) was used to provide inhalant anesthesia for lung intravital experiments only.

### 4.3. Experimental Groups

A total of 7 experimental groups (*n* = 4–10) were included in the initial study portion (2.1): NAÏVE, LPS 2H, CON 2H, LPS 4H, CON 4H, LPS 6H, and CON 6H. Groups were defined based on the following criteria. For intranasal administration CON mice received sterile saline while LPS mice received LPS solution. Tissues were collected at the designated timepoint (2 h, 4 h, or 6 h post-induction).

A total of 9 experimental groups (*n* = 5–9) were included in the latter study portion (2.2, 2.3): NAÏVE, SHAM + VEH, SHAM + HU, CON + VEH, CON + HU, LPS + VEH, LPS + HU, LPS + DEX, and LPS + DEX + HU. Groups were defined based on the following criteria. For intranasal administration, CON mice received sterile saline, LPS mice received LPS solution, and SHAM mice were anesthetized but received no intranasal administration. For intravenous treatment administration, VEH mice received vehicle only, HU mice received HU-308, DEX mice received dexamethasone, and DEX + HU mice received both dexamethasone and HU-308. NAÏVE mice underwent no induction procedures prior to experimentation. Tissue collection or IVM were performed at 6 h post-induction.

### 4.4. ALI Induction

Mice were anesthetized by intraperitoneal injection of sodium pentobarbital (27 mg/mL; 90 mg/kg) or inhalation of isoflurane (1–5%; lung IVM experiments only) through a nose cone. Once anesthetized, mice were secured by upper incisors in a 60° supine position on a small animal intubation stand (Kent Scientific; Torrington, CT, USA). The tongue was withdrawn in a cranial and lateral direction with forceps to prevent entry into the gastrointestinal tract. Small droplets of LPS were instilled into the left naris every 5–10 s to a dosage of 5 mg/kg (0.5 mL/kg, or eq. vol. sterile saline) using a 20 µL pipette (ThermoFisher Scientific; Waltham, MA, USA). This procedure was then repeated with a washout of 5.0 µL of sterile saline to increase LPS entry into lower respiratory tract. CON mice received an equivalent volume of sterile saline instead of LPS solution. SHAM mice were anesthetized but received no intranasal administration. Mice were then placed in isolated recovery cages with ad libitum access to standard rodent chow, water, and heat, and were monitored hourly pending further experimentation.

### 4.5. Treatment Administration

Treatment was administered immediately following ALI induction. A 1 mL BD tuberculin syringe fitted with a 30-gauge needle (0.3 mm × 13 m; BD, Franklin Lakes, NJ, USA) was used to administer the appropriate solution(s) via tail vein. Mice receiving vehicle only were administered 3 mL/kg of a 1:1:18 solution of anhydrous ethanol: Kolliphor EL: sterile saline. Mice receiving HU-308 only (3 mg/kg) were administered 3 mL/kg of HU-308 solution. Mice receiving dexamethasone only (0.1 mg/kg) were administered a 3 mL/kg bolus of dexamethasone solution. Mice receiving combination treatment of HU-308 and dexamethasone received two tail vein injections to a total volume of 6 mL/kg.

### 4.6. Tissue Collection

Mice were anesthetized with a lethal intraperitoneal injection of sodium pentobarbital (54.4 mg/mL). Blood samples were collected in pre-heparinized syringes via facial vein and cardiac punctures and immediately transferred to a 1 mL Eppendorf tube (Axygen MaxyClear Microtube; Axygen Inc.; Union City, CA, USA) and placed on ice. Samples were then transferred to a refrigerated centrifuge (4 °C) and spun at 1500× *g* for 15 min. Supernatant was isolated in 60 µL aliquots in clean 1.5 mL Eppendorf tubes and stored at −80 °C. To prepare histology samples, lungs were surgically extracted from the thoracic cavity and inflated with 10 mL of 10% neutral buffered formalin (NBF) via a 23-gauge blunt needle threaded into trachea. Lungs were then submerged in 10% NBF for a minimum of 24 h. Following fixation, right lungs were embedded in paraffin, cut into 5 µm sections, and stained with hematoxylin and eosin (H&E).

### 4.7. Lung Histopathology Scoring

Histopathological evidence of ALI was scored in a manner derived from previous literature [51]. Briefly, ten 400× fields of H&E-stained right lung from each mouse were randomly selected and assessed for alveolar neutrophil infiltration, interstitial neutrophil infiltration, accumulation of proteinaceous debris in the alveoli, septal thickening, and hyaline membrane formation. Scores are presented as a continuous value between 0 and 1, with 0 representing no evidence of ALI, and 1 representing severe ALI.

### 4.8. Plasma Cytokine Analysis

Plasma levels of selected inflammatory mediators (CXCL1, CXCL2, ICAM-1, IFN-γ, IL-1β, IL-6, IL-10, LIX, P-selectin, and TNFα) were analyzed using LuminexTM multiplex magnetic bead-based assays (Lot No. L136522; Bio-Techne; Minneapolis, MN, USA) according to the manufacturer’s instructions. 1:2 sample dilutions of were prepared in duplicate and read using a Bio-Rad 200 luminometer with Bio-Plex manager software.

### 4.9. Intestinal Intravital Microscopy

#### 4.9.1. Surgical Preparation

To begin the IVM procedure, mice were anesthetized by intraperitoneal injection of sodium pentobarbital (90 mg/kg). Once immobile, mice received a 2.5 mL/kg intravenous bolus of a 3:2 mixture of Rhodamine-6G and FITC-albumin via tail vein. Under deep anesthesia, mice were placed in a supine position on a heating pad and a rectal temperature probe (SomnoSuite Low-Flow Anesthesia System; Kent Scientific) was applied to monitor body temperature, which was maintained at 37.0 ± 0.5 °C for the duration of the procedure. Laparotomy was performed to access the abdominal cavity. Next, a 2–3 cm length of ileum was gently retracted from the abdominal cavity using cotton-tipped applicators moistened with warm saline. Mice were then carefully transferred to an in-house 3D-printed IVM platform in the right lateral recumbent position and the ileum was placed on the stage under an imaging grade glass cover slide. The exposed length of ileum was perfused with warm saline (37.0 °C; 5 mL/min) for the duration of the procedure, and ten minutes were allowed to pass for stabilization prior to imaging. Dilute pentobarbital (5.4 mg/mL) was added intermittently directly to the ileum as needed to maintain depth of anesthesia and control peristalsis. To surgically expose the mucosal layer with following imaging of the other anatomical regions, two ~0.5 cm parallel incisions approximately 1.5 cm apart were made transverse to the intestinal lumen so as not to sever any large vessels, and an electro-cautery device and scalpel were used to sever the intestinal wall. Finally, the mucosa was washed with saline to remove debris and fecal matter.

#### 4.9.2. Microscopy

Intravital fluorescence imaging was performed with an epifluorescence microscope (MST49; Leica Microsystems; Wetzlar, Germany) fitted with a 20× objective (N Plan L 20×/0.40 Long Working Distance Microscope Objective; Leica Microsystems) and a mercury-arc light source (LEJ EBQ 100 W; Leistungselektronik Jena GmbH; Jena, Germany). Collecting venules (V1) were identified by a vessel diameter of 60–100 µm and presence of a paired arteriole, while post-capillary venules (V3) were identified by a vessel diameter of 15–35 µm. Six fields of view (FOV) of each vessel type were recorded for 30 s using a 530–550 nm bandpass excitation filter to excite Rhodamine-6G, thereby providing visualization of leukocytes. The muscularis layer was identified by transverse and longitudinal arrangement of capillaries, while mucosal villi were identified by their distinctive arrangement after exposing the mucosal later. Six FOV of each layer were recorded for 30 s using a 460–490 nm bandpass excitation filter to excite FITC, thereby providing visualization of blood flow.

#### 4.9.3. Video Analysis

Leukocyte-endothelial interactions and FCD were analyzed in a blinded manual fashion using Fiji [52]. For leukocyte endothelial-interactions, ROI were determined by the longest unbranched portion of the vessel and endothelial surface area was estimated under the assumption of cylindrical vessel geometry. Leukocyte adhesion was defined as the number of leukocytes which remained immobile on the endothelium within the ROI for the duration of the video (30 s) and was quantified in cells/mm^2^. Leukocyte rolling was defined as the number of leukocytes which passed across an operator-defined reference line drawn perpendicular to the direction of blood flow and was quantified in cells/minute. For FCD, muscularis layer ROI were determined by the largest rectangular area which remained in focus, while mucosal layer ROI were determined by the outline of each intestinal villus which remained in focus. FCD was quantified in cm/cm^2^, where the numerator is the aggregate length of all perfused capillaries within the ROI and the denominator is the cumulative planar surface area of all ROI.

### 4.10. Lung Intravital Microscopy

#### 4.10.1. Surgical Preparation

Animals were prepared for lung IVM via thoracotomy under inhalant anesthesia (1.0–2.5% isoflurane). Mice were anesthetized, orotracheally intubated, and stabilized on a SomnoSuite Low-Flow Anesthesia System (Kent Scientific) under pressure-controlled ventilation (20 cmH_2_O) and a positive end-expiratory pressure of 5 cmH_2_O. Mice were then secured in the right lateral decubitus position on an in-house 3D-printed IVM platform. Blunt dissection was performed to expose the rib cage, and a 2.5 mL/kg intravenous bolus of a 3:2 mixture of Rhodamine-6G and FITC-albumin was administered via tail vein. The left lung was then exposed by excising a ~1 cm × 2 cm portion of the ribcage, using electrocautery to preserve hemodynamics. Finally, the left lung was stabilized for imaging via a vacuum-stabilized imaging window (Luxidea; Calgary, AB, Canada) connected to a vacuum pump (Cole-Parmer Canada; Laval, QC, Canada) set to deliver ~55 mmHg constant suction.

#### 4.10.2. Microscopy

Intravital fluorescence imaging was performed using the aforementioned microscope set-up. Pulmonary venules and arterioles were identified by convergent and divergent patterns of blood flow, respectively, while capillary ROI were identified as areas not intersected by large vessels. Five FOV of each vessel type (venules, arterioles, capillary ROI) were recorded for 30 s using the 460–490 nm bandpass excitation filter to excite FITC, followed by 30 s using the 530–550 nm bandpass excitation filter to excite Rhodamine-6G.

#### 4.10.3. Video Analysis

Leukocyte-endothelial interactions and FCD were analyzed in a blinded manual fashion using Fiji [52]. Venular and arteriolar ROI were defined using the FITC channel to identify the longest unbranched portion of each vessel and endothelial surface area was estimated under the assumption of cylindrical vessel geometry. Venular and arteriolar leukocyte adhesion was defined in the same manner previously described for intestinal IVM. Leukocyte rolling in these vessels was defined in a similar manner to intestinal IVM, with an added criteria to exclude free-flowing leukocytes by comparing speed of passage with that of red blood cell flow (due to lower flow rate of pulmonary circulation). Leukocyte adhesion in capillary ROI was defined in a similar manner to venules and arterioles but calculated under the assumption of planar geometry. FCD in capillary ROI was quantified in the same manner previously described for intestinal IVM.

### 4.11. Statistical Analysis

Statistical analysis was performed with GraphPad Prism version 9.3.1 (GraphPad Software Inc., La Jolla, CA, USA). Distribution of data was tested via Shapiro–Wilk normality test. Two-tailed Mann–Whitney tests were used to compare differences between LPS and CON groups at 2, 4, and 6 h timepoints. This data is presented as box-and-whisker plots with whiskers showing minimum and maximum values, boxes represent 25–75% data ranges, and horizontal lines within boxes represent medians. For all subsequent experiments, normally distributed data were analyzed using one-way ANOVA followed by Dunnett’s multiple comparisons test with LPS + VEH as reference group. This data is presented as mean ± standard deviation (SD). Differences were considered statistically significant where *p* < 0.05.

## 5. Conclusions

The present study investigated the effect of CB_2_ agonist HU-308 on systemic and pulmonary inflammation in an experimental murine model of pneumonia-induced ALI. We observed that HU-308 attenuated systemic inflammatory cytokine release, reduced intestinal leukocyte adhesion, and modestly reduced histological lung injury. These results suggest an attenuation of systemic inflammation with minimal impairment of pulmonary host defense. CB_2_ activation may thus demonstrate therapeutic potential to improve outcome in pneumonia-induced ALI.

## Data Availability

Not applicable.

## Supplementary Materials

The following supporting information can be downloaded at: https://www.mdpi.com/article/10.3390/ijms232415857/s1.
