# Supplementary material for: Selective CB2 Receptor Agonist, HU-308, Reduces Systemic Inflammation in Endotoxin Model of Pneumonia-Induced Acute Lung Injury
Source: Int J Mol Sci. 2022 Dec 13;23(24):15857. doi: 10.3390/ijms232415857 (PMC9779896; doi:10.3390/ijms232415857)
Supplement: Supplementary file 1 [file ijms-23-15857-s001.zip › Supplemental Figures.pdf]

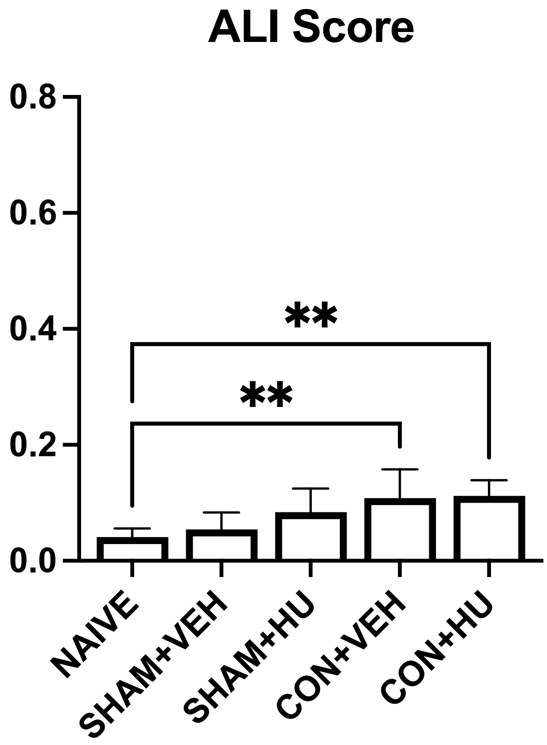

Supplemental Figure S1. Histopathological lung scores depicting impact of saline and vehicle administration on lung histopathology. Administration of saline induced a minor increase in ALI score. Y-axis ranges set based on Figure 6. Data are expressed as mean ± SD. Statistically significant differences were determined via one-way ANOVA with Dunnett's multiple comparisons test and are depicted as *p ≤ 0.05, ** p ≤ 0.01, ***p ≤ 0.001, ****p ≤ 0.0001.

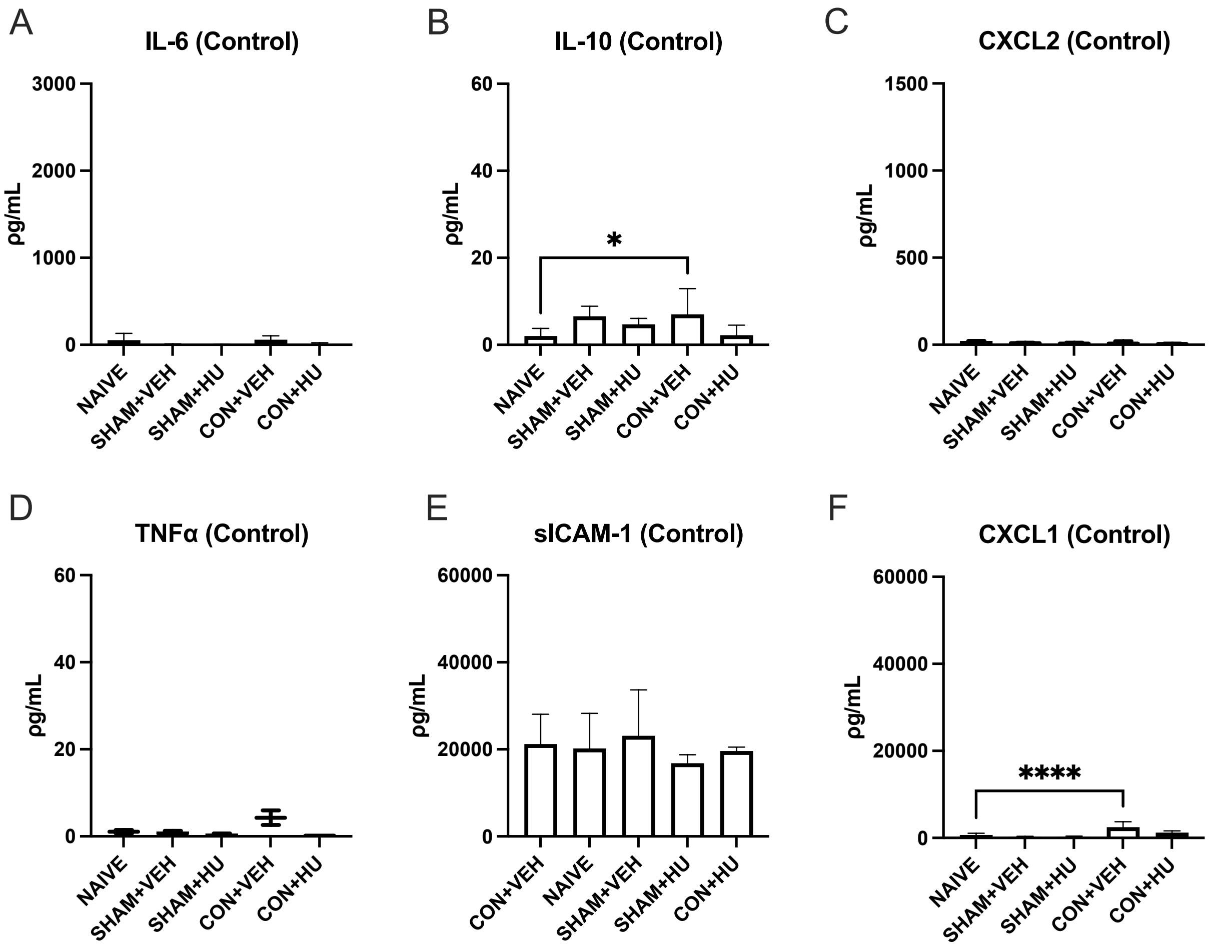

Supplemental Figure S2. Plasma cytokine levels depicting impact of saline and vehicle administration on systemic inflammatory mediator release. (A) IL-6, (C) CXCL2, (D) TNFα, (E) sICAM-1 were unaffected. (B) IL-10 and (F) CXCL1 were slightly increased versus naïve. Y-axis ranges set based on Figure 3. Normally distributed data (A, B, E, F) expressed as mean ± SD. Non-parametric data (C, D) expressed as box-and-whisker plot. Statistically significant differences were determined via one-way ANOVA (parametric data) or Kruskal-Wallis test (non-parametric data) with Dunnett's multiple comparisons test and are depicted as *p ≤ 0.05, ** p ≤ 0.01, ***p ≤ 0.001, ****p ≤ 0.0001.
