# Supplementary material for: Selective CB2 Receptor Agonist, HU-308, Reduces Systemic Inflammation in Endotoxin Model of Pneumonia-Induced Acute Lung Injury
Source: Int J Mol Sci. 2022 Dec 13;23(24):15857. doi: 10.3390/ijms232415857 (PMC9779896; doi:10.3390/ijms232415857)
Supplement: Supplementary file 1 [file ijms-23-15857-s001.zip › Supplemental Legend.pdf]

Legend for Supplemental Figures and Videos

Figure S1: ALI scores depicting impact of saline and vehicle administration on lung histopathology.

Figure S2: Plasma cytokine levels depicting impact of saline and vehicle administration on systemic inflammatory mediator release.

Video S1: Leukocyte trafficking (V1) in CON+VEH mouse.

Video S2: Leukocyte trafficking (V3) in CON+VEH mouse.

Video S3: Leukocyte trafficking (V1) in LPS+VEH mouse.

Video S4: Leukocyte trafficking (V3) in LPS+VEH mouse.

Video S5: Muscularis capillary perfusion in CON+VEH mouse.

Video S6: Mucosal capillary perfusion in CON+VEH mouse.

Video S7: Muscularis capillary perfusion in LPS+VEH mouse.

Video S8: Mucosal capillary perfusion in LPS+VEH mouse.

Video S9: Leukocyte trafficking in pulmonary venule of CON+VEH mouse.

Video S10: Leukocyte trafficking in pulmonary venule of LPS+VEH mouse.

Video S11: Leukocyte trafficking in pulmonary arteriole of CON+VEH mouse.

Video S12: Leukocyte trafficking in pulmonary arteriole of LPS+VEH mouse.

Video S13: Leukocyte trafficking in pulmonary capillaries of CON+VEH mouse.

Video S14: Leukocyte trafficking in pulmonary capillaries of LPS+VEH mouse.

Video S15: Pulmonary capillary perfusion in CON+VEH mouse.

Video S16: Pulmonary capillary perfusion in LPS+VEH mouse.
